# PAF and Haematopoiesis. I. 5-Fluoro-Uracil Induces PAF Production in Haematopoietic Organs of Rats

**DOI:** 10.1155/S0962935194000049

**Published:** 1994

**Authors:** Y. Denizot, V. Praloran

**Affiliations:** Laboratoire d'Hématologie Expérimentale Faculté de Médecine 2 rue du Dr. Marcland Limoges 87025 France

## Abstract

Haematopoietic organs of rats were examined for the presence of platelet-activating factor (PAF) and acetylhydrolase before and after treatment with 5-fluoro-uracil (5-FU) (200 mg/kg) a chemotherapeutic compound with apoptotic effects. PAF was reported in thymus, spleen and femoral bone marrow of rats with or without 5-FU. Although acetylhydrolase activity in organs was not affected by 5-FU treatment, elevated levels of PAF were observed in thymus and spleen. For the first time PAF is reported in haematopoietic organs of rats, strengthening *in vitro* data suggesting its role in the apoptotic processes in thymus, in the modulation of the immune response, and in the regulation of haematopoiesis.


Mediators of Inflammation 3, 23–25 (1994)

### PAF and haematopoiesis. I. 5-Fluoro-uracil induces PAF production in haematopoietic organs of rats

Y. Denizot[CA] and V. Praloran

Laboratoire d'Hématologie Expérimentale, Faculté de Médecine, 2 rue du Dr. Marcland, 87025 Limoges, France

[CA] Corresponding Author

**HAEMATOPOIETIC organs of rats were examined for the presence of platelet-activating factor (PAF) and acetylhydrolase before and after treatment with 5-fluoro-uracil (5-FU) (200 mg/kg) a chemotherapeutic compound with apoptotic effects. PAF was reported in thymus, spleen and femoral bone marrow of rats with or without 5-FU. Although acetylhydrolase activity in organs was not affected by 5-FU treatment, elevated levels of PAF were observed in thymus and spleen. For the first time PAF is reported in haematopoietic organs of rats, strengthening *in vitro* data suggesting its role in the apoptotic processes in thymus, in the modulation of the immune response, and in the regulation of haematopoiesis.**

**Key words:** 5-Fluoro-uracil, Acetylhydrolase, Platelet-activating factor, Spleen, Thymus

### Introduction

Platelet-activating factor (PAF, 1-*O*-alkyl-2-acetyl-*sn*-glycero-3-phosphocholine) is a phospholipid mediator of inflammation which is produced from and activates a wide variety of inflammatory cells such as polymorphonuclear neutrophils and monocytes.[1] Regulating PAF levels is of importance since elevated levels of PAF could result in pathological effects. Removal of the acetyl moiety of PAF abolishes its biological activity.[2] A specific acetylhydrolase, naturally present in blood and tissues, regulates PAF concentrations.[3,4] PAF exhibits potent immunoregulatory effects on T and B cell growth and functions.[5–11] PAF enhances the DNA synthesis in guinea-pig bone marrow cells[12] and has been detected in human thymus.[13] Although data have suggested immune regulation by PAF *in vitro*, the absence of assessment of PAF in haematopoietic organs may cast some doubts about the reality of its putative physiological role *in vivo*. Thus the authors have examined PAF levels and acetylhydrolase activity in haematopoietic organs of rats before and after depletion of haematopoietic proliferative cells with 5-fluoro-uracil (5-FU), a potent chemotherapeutic drug with apoptotic effects.[14]

### Materials and Methods

*Animals:* Normal adult female Wistar rats weighing 300–400 g were housed in two groups of five. One group was given an intraperitoneal injection of 5-FU (200 mg/kg). Sixty hours later all the animals were killed by cervical dislocation.

*Processing of samples for PAF determinations:* Half the thymus, half the spleen, and a part of the liver were weighed, minced into small pieces, washed in saline, and homogenized in 4 ml of ethanol. One ml of blood was mixed with 4 ml of ethanol. The bone marrow was flushed from a femur with 2 ml of ethanol. Lipids from all samples were extracted with ethanol for 10 h at 4°C. Phospholipids were purified by thin layer chromatography (TLC).[15] Areas of samples on TLC plates with $R_F$ values corresponding to a PAF synthetic standard were extracted, and assayed for PAF activity.

*Processing of samples for acetylhydrolase measurements:* Half the thymus, half the spleen, and a part of the liver were weighed, minced into small pieces, washed in saline, and homogenized in 2 ml of Tyrode's buffer at 4°C. Serum samples were recovered from blood. The bone marrow was flushed from a femur with 2 ml of Tyrode's buffer. Samples were centrifuged and supernatants were stored at −80°C until assay of the PAF acetylhydrolase activity.

*PAF assay:* Washed rabbit platelets were prepared as described previously.[16] Aspirin-treated platelets in Tyrode's buffer containing the adenosine diphosphate scavenger mixture, creatine phosphate (1 mM)/creatine phosphokinase (10 U/ml) were stirred in an aggregometer (Labintec, France). Aggregating activity of the samples was measured using a calibration curve obtained with 2.5 to 50 pg synthetic PAF (Novabiochem, Switzerland).

*PAF acetylhydrolase assay:* For the assay, supernatants were tested essentially as described previously.[3,16] Results were expressed in nM of PAF degraded per min per g (wet weight) of femur, or ml of serum, as means of duplicate determinations. The variation between duplicates was less than 3%.

© 1994 Rapid Communications of Oxford Ltd

*Statistical analysis:* Data are shown as mean ± S.E.M. of five animals. Normality of data distribution was assessed using the Kolmogorov Smirnov's test. Means were compared using Student's *t*-test for paired variables. A $p < 0.05$ was considered significant.

### Results

Lipids were extracted from the spleen, thymus, liver, blood and femoral bone marrow. After purification by TLC, PAF was assessed by platelet aggregation. As shown in Fig. 1, PAF was found in spleen, thymus and liver. PAF was detected in bone marrow (0.25 ± 0.04 ng PAF/femur). Minute amounts of PAF were assessed in blood (0.02 ± 0.01 ng PAF/ml). In control rats PAF levels per g (wet weight) were six-fold and two-fold greater in spleen and thymus than in liver ($p = 0.03$). In controls acetylhydrolase activity is significantly higher in spleen than in liver and thymus ($p = 0.008$ and $p = 0.002$, respectively), and in liver than in thymus ($p = 0.001$) (Table 1). A weak acetylhydrolase activity was docmented in bone marrow. The enzyme activity in blood was similar to that given in a previous report.[17]

Acetylhydrolase activities were not significantly different in rats with or without 5-FU (Table 1). After 5-FU, elevated levels of PAF per g (wet weight) were documented in thymus and spleen ($p > 0.05$) but not in liver ($p > 0.1$) (Fig. 1). PAF amounts in blood (0.04 ± 0.02 ng PAF/ml and in bone marrow (0.13 ± 0.01 ng PAF/femur) were not significantly different when compared with control values ($p > 0.1$).

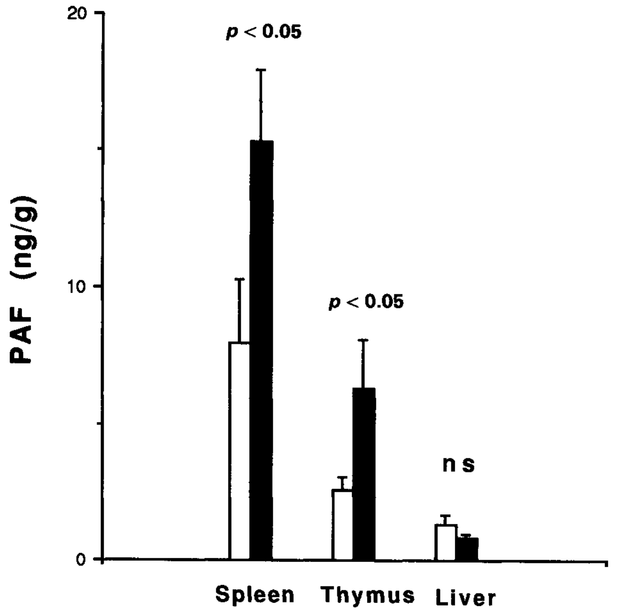

FIG. 1. PAF levels in spleen, thymus and liver of control and 5-FU-treated rats. PAF was expressed as ng PAF per g of wet weight. Mean ± S.E.M. of five animals. ns: no significant difference.

**Table 1.** Acetylhydrolase activity in spleen, thymus, liver, femoral bone marrow and serum on control and 5-FU treated rats

| Organ | Acetyl hydrolase activity: Controls | Acetyl hydrolase activity: After 5-FU treatment | *p* |
|---|---|---|---|
| Thymus | 33.8 ± 3.0 | 57.6 ± 12.1 | 0.06 |
| Spleen | 148.2 ± 19.9 | 174.0 ± 21.6 | 0.25 |
| Liver | 73.0 ± 1.1 | 80.0 ± 6.1 | 0.12 |
| Bone marrow* | 3.9 ± 0.3 | 4.3 ± 0.4 | 0.3 |
| Serum** | 97.8 ± 13.5 | 66.47 ± 8.9 | 0.09 |

Acetylhydrolase was expressed as nmol PAF degraded per min per g of wet weight. *in nmol PAF per min per femur; **in nmol PAF per min per ml serum. Mean ± S.E.M. of five animals.

Treatment with 5-FU significantly decreased the weight of the spleen (0.58 g ± 0.02 *vs.* 1.04 g ± 0.08, $p < 0.003$) but not of the thymus (0.48 g ± 0.1 *vs.* 0.54 g ± 0.08, $p = 0.13$). As previously shown this decrease is due to the disappearance of cycling cells killed by 5-FU treatment.[18]

Chemical characterization of PAF was based on several criteria including its TLC behaviour analogous to that of authentic PAF; the loss of its biological activity after treatment by phospholipase $A_2$ but not by lipase $A_1$ from *Rhizopus arrhizus*; and the inhibition of the platelet aggregation induced by organ-extracted PAF with the PAF antagonist CV 3988 (Takeda Chemical Ind., Osaka, Japan) (data not shown).

### Discussion

Numerous studies have reported the immunoregulatory functions of PAF *in vitro*.[5–11] However, the absence of data reporting the presence of PAF in haematopoietic organs may cast some doubts about its putative role *in vivo*. The authors documented PAF in bone marrow, spleen and thymus of rats, strengthening the hypothesis that PAF could play a role in the modulation of the immune response *in vivo*. Because only minute amounts of PAF are detected in blood, the PAF documented in spleen and thymus is probably linked to a local PAF production. PAF is also present in bone marrow (0.25 ng PAF per femur) suggesting that PAF could interfere with the process leading to haematopoiesis. Kato *et al.*[12] have reported that 55 to 550 pg of a non-metabolizable PAF agonist enhanced the DNA synthesis in guinea-pig bone marrow cells, indicating that the levels of PAF that were detected in femoral bone marrow could be of a physiological significance. Clearly the presence of PAF in human bone marrow deserves to be investigated.

After 5-FU treatment similar levels of PAF were noted in blood, liver and femoral bone marrow. In contrast, elevated levels of PAF were documented in spleen and thymus. Because 5-FU is known to affect cells in proliferation,[18] the present data suggest that PAF in spleen and thymus may originate from non-lymphoid populations such as macrophages, epithelial cells, and/or dendritic cells rather than B or T cells. Similarly in bone marrow PAF may originate from stromal cells which are the major component of the haemopoietic microenvironment rather than progenitor cells. The hypothesis of a PAF production from non-lymphoid populations in thymus has been suggested by Salem *et al.*,[13] who reported that CD2 negative cells in the human thymus were far more efficient than CD2 positive cells (i.e. thymocytes) in producing PAF. Furthermore even though PAF production has been reported from human lymphoid leukaemic cells[19,20] and from a human $CD4^+$ T lymphocyte clone,[21] the release of PAF from normal human lymphocytes has been unsuccessful.[22,23] At present it is unclear whether the absence of data showing PAF production from haematopoietic precursor cells is due to a paucity of studies, or to the fact that negative results have not been published. Strengthening the putative role of PAF on these immature cells, Saito *et al.*[24] have reported that PAF induced eosinophilic and basophilic differentiation in human haematopoietic precursor cells.

Because acetylhydrolase activity in thymus and spleen did not significantly vary after 5-FU treatment, the elevated amounts of PAF documented in thymus and spleen seem related to an enhanced PAF production. The molecular signal(s) and the cell type(s) implicated in PAF production in thymus and spleen are currently unknown. Anticancer drugs such as 5-FU have been reported to induce apoptosis in cancer cells and in thymocytes.[14] Thus, regardless of the origin of PAF in thymus, its function could be of importance since data have shown that, in association with another signal, PAF could modulate apoptotic processes in an immature T-cell line.[25]

It is still difficult to evaluate the physiological role of PAF *in vivo* in haematopoietic organs such as bone marrow, spleen and thymus. However, its presence strengthens the *in vitro* data which show that PAF may participate in the mechanism of programmed cell death and is a modulator of cellular immune responses.

### References

1. Benveniste J. Paf-acether, an ether phospholipid with biological activity. In: Karnovsky ML, Leaf A, Bolis LC, eds. *Biological Membranes: Aberrations in membrane structure and function.* New York: AR Liss Inc., 1988; 73–87.
2. Prescott SM, Zimmerman GA, McIntyre TM. Platelet-activating factor. *J Biol Chem* 1990; **265**: 17381–17384.
3. Miwa M, Miyake T, Yamanaka T, *et al.* Characterization of serum platelet-activating factor (PAF) acetylhydrolase. Correlation between deficiency of serum PAF acetylhydrolase and respiratory symptoms in asthmatic children. *J Clin Invest* 1988; **82**: 1983–1991.
4. Stafforini DM, McIntyre TM, Carter ME, Prescott SM. Human plasma platelet-activating factor acetylhydrolase: association with lipoprotein particles and role in the degradation of platelet-activating factor. *J Biol Chem* 1987; **262**: 4215–4222.
5. Rola-Pleszczynski M, Pouliot C, Turcotte S, Pignol B, Braquet P, Bouvrette L. Immune regulation by platelet-activating factor. I. Induction of suppressor cell activity in human monocytes and $CD8^+$ T cells and of helper activity in $CD4^+$ T cells. *J Immunol* 1988; **140**: 3547–3552.
6. Dulioust A, Duprez V, Pitton C, *et al.* Immunoregulatory functions of paf-acether. III. Down-regulation of $CD4^+$ T cells high-affinity IL-2 receptor expression. *J Immunol* 1990; **144**: 3123–3129.
7. Deryckx S, De Wall Malefyt R, Gauchat JF, Vivier E, Thomas Y, De Vries JE. Immunoregulatory functions of paf-acether. VIII. Inhibition of IL-4-induced human IgE synthesis *in vitro*. *J Immunol* 1992; **148**: 1465–1470.
8. Rola-Pleszczynki M, Pignol B, Pouliot C, Braquet P. Inhibition of human lymphocyte proliferation and interleukin 2 production by platelet-activating factor (paf-acether): reversal by a specific antagonist, BN 52021. *Biochem Biophys Res Commun* 1987; **142**: 754–760.
9. Dulious A, Vivier E, Salem P, Benveniste J, Thomas Y. Immunoregulatory functions of paf-acether. I. Effect of paf-acether on $CD4^+$ cell proliferation. *J Immunol* 1988; **140**: 240–245.
10. Vivier E, Deryckx S, Wang JL, *et al.* Immunoregulatory functions of paf-acether. VI. Inhibition of T cell activation via CD3 and potentiation of T cell activation via CD2. *Int Immunol* 1990; **2**: 545–553.
11. Leprince C, Vivier E, Treton D, *et al.* Immunoregulatory functions of paf-acether. VI. Dual effect of human B cell proliferation. *Lipids* 1991; **26**: 1204–1208.
12. Kato T, Kudo I, Hayashi H, Onozaki K, Inoue K. Augmentation of DNA synthesis in guinea pig bone marrow cells by platelet-activating factor (PAF). *Biochem Biophys Res Commun* 1988; **157**: 563–568.
13. Salem P, Denizot Y, Pitton C *et al.* Presence of paf-acether in human thymus. *FEBS Lett* 1989; **257**: 49–51.
14. Sen S, D'Incalci M. Apoptosis. Biochemical events and relevance to cancer chemotherapy. *FEBS Lett* 1992; **307**: 122–127.
15. Eliakim R, Karmeli F, Razin E, Rachmilewitz D. Role of platelet-activating factor in ulcerative colitis. Enhanced production during active disease and inhibition by sulfasalazine and prednisolone. *Gastroenterology* 1988; **95**: 1167–1172.
16. Denizot Y, Chaussade S, Nathan N, *et al.* Paf-acether and acetylhydrolase in stool of patients with Crohn's disease. *Dig Dis Sci* 1992; **37**: 432–437.
17. Caplan SM, Sun XM, Hsueh W. Hypoxia causes ischemic bowel necrosis in rats: the role of platelet-activating factor. *Gastroenterology* 1990; **99**: 979–986.
18. Hodgson GS, Bradley TR. Properties of haematopoietic stem cells surviving 5-fluoro-uracil treatment: evidence for a pre-CFU-S cell? *Nature* 1979; **281**: 381–382.
19. Bussolino F, Foa R, Malavasi F, Ferrando ML, Camussi G. Release of platelet-activating factor (PAF)-like material from human lymphoid cell lines. *Exp Hematol* 1984; **12**: 688–693.
20. Foa R, Bussolino F, Ferrando ML *et al.* Release of platelet-activating factor in human leukemia. *Cancer Res* 1985; **45**: 4483–4485.
21. Le Gouvello S, Vivier E, Debre P, Thomas Y, Colard O. CD2 triggering stimulates the formation of platelet-activating factor-acether from alkyl-arachidonyl-glycerophosphocholine in a human $CD4^+$ T lymphocyte clone. *J Immunol* 1992; **149**: 1289–1293.
22. Camussi G, Aglietta M, Coda R, Bussolino F, Piacibello W, Tetta C. Release of platelet-activating factor (PAF) and histamine. II. The cellular origin of human PAF: monocytes, polymorphonuclear neutrophils and basophils. *Immunology* 1981; **42**: 191–199.
23. Jouvin-Marche E, Ninio E, Beaurain G, Tence M, Niaudet P, Benveniste J. Biosynthesis of paf-acether (platelet-activating factor). VII. Precursors of paf-acether and acetyl-transferase activity in human leukocytes. *J Immunol* 1984; **133**: 892–898.
24. Saito H, Hayakawa T, Mita H, Akiyama K, Shida T. Paf-induced eosinophilic and basophilic differentiation in human haematopoietic precursor cells. *J Lipid Med* 1992; **5**: 135–137.
25. El Azzouzi B, Jurgens P, Benveniste J, Thomas Y. Immunoregulatory functions of paf-acether. IX. Modulation of apoptosis in an immature T cell line. *Biochem Biophys Res Commun* 1993; **190**: 320–324.

**Received 15 September 1993; accepted 21 October 1993**
